# Introduction and expression of *PIK3CA*^E545K^ in a papillary thyroid cancer *BRAF*^V600E^ cell line leads to a dedifferentiated aggressive phenotype

**DOI:** 10.1186/s40463-022-00558-w

**Published:** 2022-02-22

**Authors:** Nicole Pinto, Kara M. Ruicci, Mohammed Imran Khan, Mushfiq Hassan Shaikh, Yu Fan Peter Zeng, John Yoo, Kevin Fung, S. Danielle MacNeil, Adrian Mendez, Joe S. Mymryk, John W. Barrett, Paul C. Boutros, Anthony C. Nichols

**Affiliations:** 1 Department of Otolaryngology – Head and Neck Surgery, Western University, London, ON Canada; 2 Department of Anatomy and Cell Biology, Western University, London, ON Canada; 3 Department of Oncology, Western University, London, ON Canada; 4 London Regional Cancer Program, Lawson Health Research Institute, London, ON Canada; 5 Department of Microbiology and Immunology, Western University, London, ON Canada; 6 Eli and Edythe Broad Center of Regenerative Medicine and Stem Cell Research, University of California, Los Angeles, USA; 7 Institute for Precision Health, University of California, Los Angeles, USA; 8 Jonsson Comprehensive Cancer Centre, University of California, Los Angeles, USA; 9 London Health Sciences Centre, 800 Commissioners Rd. E., P.O. Box 5010, STN B, London, ON N6A 5W9 Canada

**Keywords:** Papillary thyroid cancer, Anaplastic thyroid cancer, Disease progression, Dedifferentiation

## Abstract

Anaplastic thyroid cancer (ATC) is a rare, aggressive form of undifferentiated thyroid cancer, which exhibits rapid progression and is almost universally fatal. At least a subset of ATC is thought to arise from pre-existing well-differentiated thyroid cancer, most frequently papillary thyroid cancer (PTC). While *PIK3CA* mutations are rare in PTC, they are common in ATC and tend to co-occur with *BRAF* mutations. This provided the rationale for our study to identify the potential role of *PIK3CA* mutations in the progression from well-differentiated to undifferentiated thyroid cancer. We introduced *PIK3CA*^E545K^ into the LAM1 PTC cell line, which carries a *BRAF*^V600E^ mutation. In culture, the engineered cell line (LAM1:*PIK3CA*^E545K^) proliferated faster and demonstrated increased clonogenic potential relative to the parental line carrying an empty vector (LAM1^EV^). Both the LAM1^EV^ and LAM1:*PIK3CA*^E545K^ edited lines were implanted into hind flanks of athymic nude mice for in vivo determination of disease progression. While tumour weights and volumes were not significantly higher in LAM1:*PIK3CA*^E545K^ mice, there was a decrease in expression of thyroid differentiation markers TTF-1, thyroglobulin, PAX8 and B-catenin, suggesting that introduction of *PIK3CA*^E545K^ led to dedifferentiation in vivo. Collectively, this study provides evidence of a role for *PIK3CA*^E545K^ in driving disease progression from a well-differentiated to an undifferentiated thyroid cancer; however, over-expression was not a determinant of an accelerated growth phenotype in ATC.

**Graphical Abstract:**

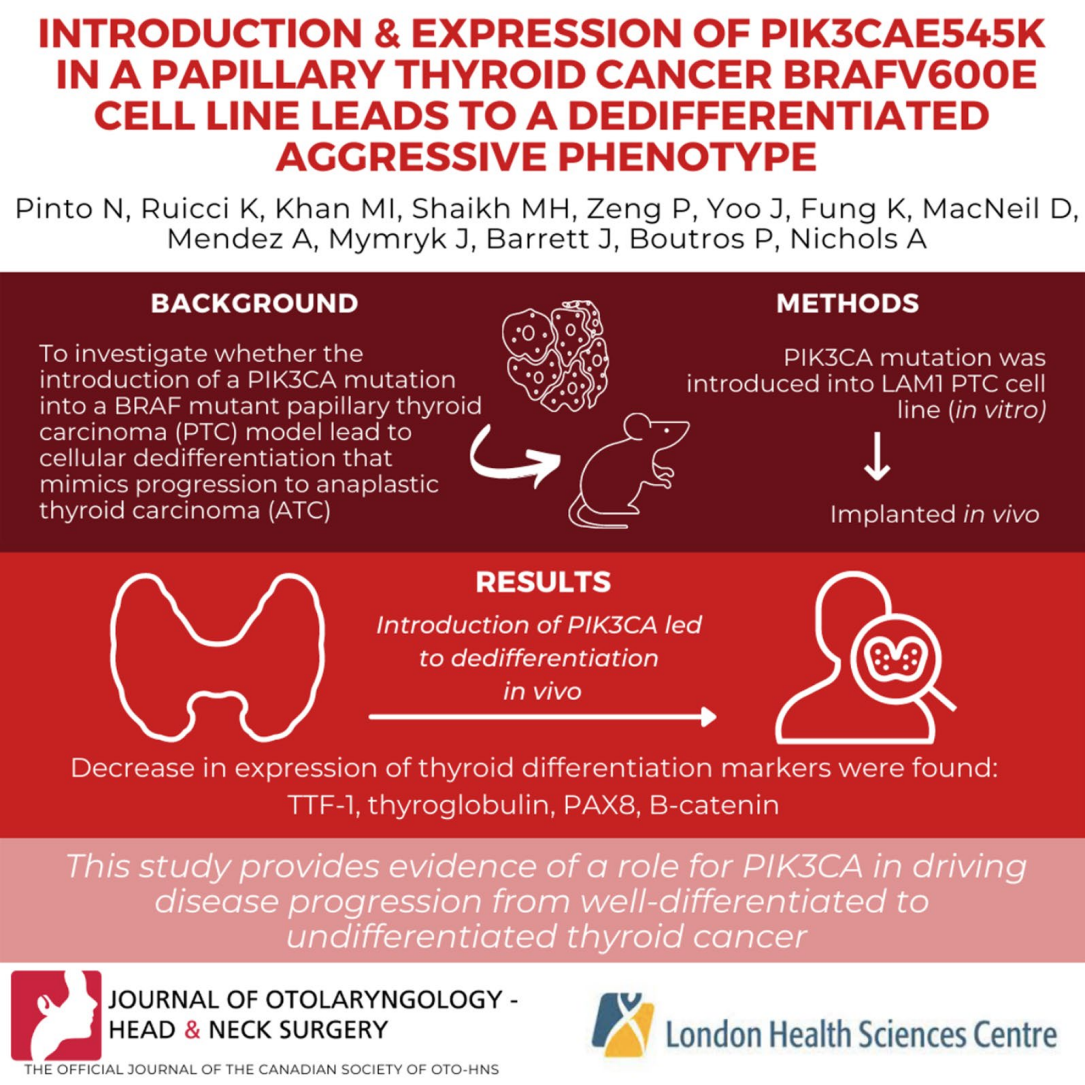

**Supplementary Information:**

The online version contains supplementary material available at 10.1186/s40463-022-00558-w.

## Introduction

Well-differentiated subtypes of thyroid cancer (papillary thyroid cancer, PTC; follicular thyroid cancer; FTC) have an excellent prognoses, typically with greater than 95% five-year survival rates [1]. In contrast, anaplastic thyroid cancer (ATC) is a rare, undifferentiated thyroid cancer which progresses rapidly and has a mortality rate of over 90% [2, 3]. Surgery, radiation and chemotherapy are often futile for ATC, with patients experiencing a median survival of approximately six months following diagnosis and some patients dying within days of diagnosis [4–7]. Thus, ATC represents one of the most lethal human malignancies.

PTC has a low frequency of genetic alterations, with the majority of tumours carrying a single driver mutation and an absence of tumour suppressor alterations [8]. In contrast, the mutational landscape of ATC is more complex, with many tumours carrying mutations in multiple oncogenes in addition to tumour suppressors [9]. One current model of thyroid carcinoma suggests that ATC arises from pre-existing well-differentiated thyroid cancer (WDTC) following accumulation of additional genetic events, producing a much more aggressive cancer [2]. Indeed, up to 79% of patients diagnosed with ATC have a co-existing region or a prior history of WDTC (most frequently PTC), providing further evidence of its derivation from pre-existing thyroid cancer lesions [10]. One particular cluster of ATC tumours carried both *BRAF*^V600E^ and the *PIK3CA* canonical hotspot mutations, with the E545K variant being the most common [9]. While *BRAF*^V600E^ mutations have a very high frequency in both PTC and ATC tumours (59.7% and 45%, respectively), mutational changes in *PIK3CA* are only present in 0.5% of PTC patient samples and at a much higher frequency of 18% in ATC [8, 9]. As *BRAF* and *PIK3CA* mutations tended to co-occur in the ATC tumours, we hypothesized that the introduction of a *PIK3CA* mutation into a *BRAF* mutant PTC model would lead to increased aggressiveness and cellular dedifferentiation, mimicking a possible route of progression from PTC to ATC.

## Materials and methods

### Cell lines and culture conditions

The LAM1 cell line, a gift from Dr. John Copland (Mayo Clinic, Jacksonville, Florida) which is a confirmed *BRAF*^V600E^ PTC cell line, was used for this study [11]. The LAM1 cell line was cultured in DMEM/F12 media supplemented with 10% FBS (GIBCO), penicillin (100 µg/mL) and streptomycin (100 µg/mL) (Invitrogen). Short tandem repeat (STR) profiling was carried out at The Centre for Advanced Genomics as described previously [12]. PCI6A and JHU029 are both head and neck squamous cell cancer cell lines and both carry canonical hot spot mutations in *PIK3CA*. PCI6A carries an E545K mutation and JHU029 has a H1047L. For the purpose of this study, PCI6A was used as a positive control for the expression of the E545K mutation and JHU029 was used to confirm the specificity of the mutant primer qPCR.

### Transfection of a PIK3CA^E545K^ expression vector into the LAM1 cell line

The most frequent *PIK3CA* variant occurring in ATC is the E545K variant [9], providing the rationale for our study. PIK3CA exon9 E545K was a gift from Bert Vogelstein (Addgene plasmid #16,642). This expression vector [13] was used to transfect LAM1 parental cell**s** (PTC *BRAF*^V600E^) to generate a stable *PIK3CA*^E545K^ mutant for use in over-expression studies. The mutant cell line was denoted as LAM1:*PIK3CA*^E545K^. A parallel transfection was performed using an empty vector control plasmid (denoted as LAM1^EV^). The parental cell line was transfected using Lipofectamine 3000 Reagent (Cat no. L3000001, Thermo Fisher Scientific) according to the standard protocol using 5 µg of plasmid DNA. Cells were incubated overnight, and transfection media was replaced the following day with normal cell line media. The following day, geneticin (G418) was added for selection at a concentration of 300 µg/mL to select against untransfected cells. The concentration of G418 used was predetermined for the LAM1 cell line using a standard kill curve.

### Immunoblotting

Immunoblotting was completed as described previously with 20 µg protein loaded per well (Pinto et al*.* 2018). PI3 kinase (p110α) (Cat no. 4249) and α-tubulin (Cat no. 2125) antibodies were obtained from Cell Signaling Technology. Membranes were incubated with peroxidase-conjugated anti-rabbit secondary antibodies diluted 1:5000 for 1 h at room temperature. Detection of target proteins was performed using Luminata Forte Western HRP substrate (EMD Millipore). α-tubulin was used as a loading control.

### Growth curves

Cells were seeded at a density of 2400 cells per well into 96-well plates. Plates were then read using PrestoBlue (Thermo Fisher Scientific) at 0, 24, 48 and 72 h. Fluorescence readings were completed using a BioTek Synergy Microplate Reader with 560 nm excitation and 590 nm emission wavelengths. An unpaired Student’s, two-tail *t*-test was used for statistical analysis, where comparisons were made between LAM1^EV^ and LAM1:PIK3CA^E545K^ cell lines for each time point. *P* values < 0.05 were considered to represent statistically significant differences. Statistical analysis is displayed for the 72-h time point when compared to the control at the same time point.

### Dose–response curves (Etoposide)

Etoposide is a chemotherapy used clinically to treat ATC, and functions by inhibiting the enzyme topoisomerase II. Cell lines were seeded into 96-well plates at a density of 2400 cells per well and incubated overnight at 37 °C and 5% CO_2_. After 24 h, cells were treated with Etoposide (Cat no E1383; Sigma Aldrich) using a concentration range of 0.125 to 32 µM. After 72 h, plates were read by incubating cells with PrestoBlue for 1 h at 37 °C. After incubation with PrestoBlue, fluorescence readings were completed using a BioTek Synergy Microplate Reader with 560 nm excitation and 590 nm emission wavelengths. To generate dose–response curves, raw fluorescence data was loaded into Prism® 8 GraphPad Software, where the values were then normalized to the untreated control samples and the average viability for each concentration tested was calculated. In order to determine the half-maximal inhibitory concentration (IC_50_), the normalized relative fluorescence units (RFU) of the Etoposide-treated samples were calculated as a percentage of the mean RFU of the untreated samples. Drug concentrations were then transformed to a logarithmic scale (Log_10_) and IC_50_ values were calculated using non-linear regression (curve fit) (Prism8).

### Clonogenic assay

LAM1^EV^ and LAM1:*PIK3CA*^E545K^ cells were seeded into six-well tissue culture plates at a density of 1,000 cells per well. Cells incubated at 37 °C for 10 days based on colony formation of the parental cells with cell media changed every three days. Cells were washed with PBS and fixed with 100% chilled methanol for 15 min. Following fixation, cells were stained with 0.5% crystal violet in 10% methanol/1 × PBS for a total of 10 min. Brightfield microscopy was used to quantify colony formation, with a colony defined as being ≥ 50 cells. For quantification, three representative fields per well were counted for the number of colonies and this was completed for both the parental and mutant cell lines. LAM1:*PIK3CA*^E545K^ cells were compared to the parental control line and *p* values < 0.05 were considered to represent statistically significant differences in colony formation (Student’s t-test).

### Clonogenic assay: radiation response

LAM^EV^ and LAM1:*PIK3CA*^E545K^ cells were seeded at a density of 250 (0 Gy), 500 (1 Gy), 1000 (2 Gy) and 2000 (4 Gy) cells per well in six-well plates, with respective doses of radiation applied after 24 h (with three biological replicates). Cells were incubated at 37 °C for a one-week period with cell media changed every three days. Cells were washed with PBS and fixed with 100% chilled methanol for 15 min. Following fixation, cells were stained with 0.5% crystal violet in 10% methanol/1 × PBS for 10 min. Stained colonies were quantified and counted using brightfield microscopy with a colony defined as being ≥ 50 cells. The plating efficiency (PE) of cells that were untreated was calculated. The survival fraction was calculated and defined as the number of colonies that arose following radiation treatment, and expressed in terms of PE [14].

### Cell migration assay

Cell migration for LAM1^EV^ and LAM1:*PIK3CA*^E545K^ cell lines were quantified using a 24-well cell migration plate and fluorometric analysis (Cat no. CBA-100; Cell Biolabs Inc). The initial cell suspension was placed into the upper chamber containing serum free media at a density of 5 × 10^4^ cells per chamber, with three biological replicates. Cells were incubated at 37 °C to allow migratory cells to pass through the polycarbonate membrane and cling to the bottom of the chamber. Cells that stayed in the upper chamber were considered to be non-migratory. Migratory cells were then dissociated from the membrane of the chamber using the Cell Detachment Buffer provided in the cell migration plate kit. Cells which migrated through were lysed and quantified using CyQuant GR Fluorescent Dye provided in the same kit. Fluorescence readings were completed using a BioTek Synergy Microplate Reader and representative images were collected using brightfield microscopy.

### Hind flank model to study thyroid cancer disease progression

Mice were maintained and handled in accordance with the Western University AUP 2015–045 protocol. We used the cell lines LAM1^EV^ and LAM1:*PIK3CA*^E545K^ for the generation of cell line xenograft hind flank models of disease (*n* = 5 per group). Cell lines were treated with plasmocin for two weeks prior to use in mouse models and screened for mycoplasma before use. Both cell lines were cultured in DMEM/F12 media supplemented with 10% heat inactivated FBS without antibiotics for two weeks before initiation of experimentation. A total of 1 × 10^6^ cells were injected subcutaneously into the hind flank of each athymic nude mouse. Mice were weighed twice weekly and tumour dimensions (length and width) were measured once weekly when tumours were palpable using digital calipers. Individual tumour volumes were calculated using the formula: [length × (width)^2^] × 0.52 [15]. Mice were sacrificed at 14 weeks post-injection. Tumours were dissected, weights were recorded in milligrams (mg) (Additional file 2: Table S2) and stored in formalin for FFPE block preparation and IHC studies.

### Immunohistochemistry

Immunohistochemistry (IHC) staining was carried out in the Molecular Pathology Core Facility (Western University) where 4-µM sections were cut and tested for expression of thyroid-related markers. Primary antibodies were used according to the manufacturer’s instructions: TTF-1 (Abcam; ab76013), PAX8 (Abcam; ab76013), Beta Catenin (Cell Signalling; 8480S), Ki67 (Cell Signalling; 9027S) and Thyroglobulin (Agilent; IR50961-2). Slides were scanned using an Aperio ScanScope slide scanner.

### Statistical analysis

A Student’s unpaired, two-tail *t*-test was performed using Prism® 8 Graphpad Software Macintosh Version (by Software MacKiev © 1994–2014 GraphPad Software, Inc). *P* values < 0.05 were considered statistically significant. *P* values are defined as not significant *p* > 0.05, * represents *p* < 0.05, ** represents *p* < 0.01, *** represents *p* < 0.001 and **** represents *p* < 0.0001.

## Results

### Transfection of PIK3CA^E545K^ into LAM1 cells promoted increased cellular proliferation, colony formation and cell migration

**Fig. 1:**
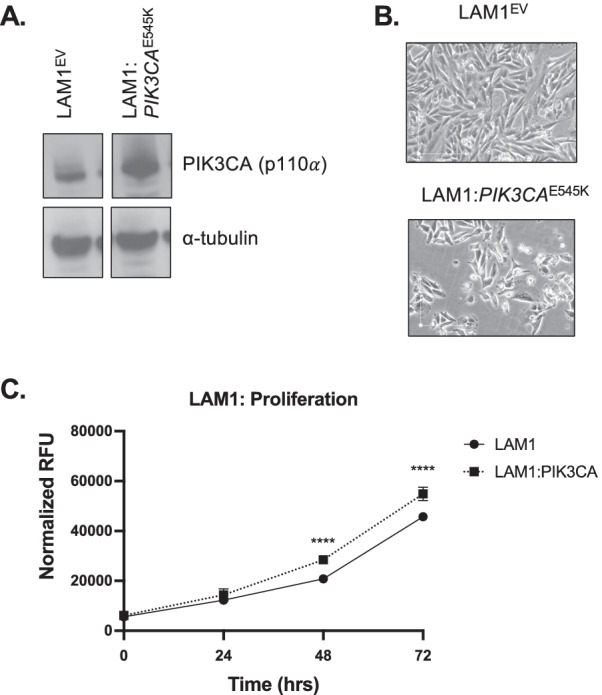
Expression of activated *PIK3CA*^E545K^ mutation into a *BRAF*-mutant PTC cell line leads to an increase in cell proliferation. **A** Immunoblot analysis of the LAM1 cell line containing the *BRAF*^V600E^-mutant with an empty vector (LAM1^EV^) and the LAM1 cell line with the *PIK3CA*^E545K^ added (LAM1:*PIK3CA*^E545K^). Cells were collected and whole cell lysates were prepared with 20 µg total protein loaded per well. α-tubulin was used as a loading control. **B** Brightfield microscopy images were collected 24 h after plating of the LAM1^EV^ cell line and the LAM1:*PIK3CA*^E545K^ cell lines. Cells were seeded at a density of 150,000 cells per well. **C** Cells were seeded at a density of 2400 cells per well and incubated for 24 h. Cellular proliferation was measured with the addition of PrestoBlue to cells, which were then incubated for one hour prior to plate readings. Relative Fluorescent Units (RFU) on the y-axis represent the fluorescence of the PrestoBlue output. Changes in cellular proliferation were measured at baseline, 24, 48 and 72 h for both LAM1^EV^ and LAM1:*PIK3CA*^E545K^ cell lines

To study the progression of a thyroid cancer from a well-differentiated to an undifferentiated cell state, we started with the human PTC cell line LAM1, which contains the *BRAF*^V600E^ mutation found in a significant fraction of ATCs [16]. LAM1 cells were transfected to stably express *PIK3CA*^E545K^, which is also commonly found in ATC [9]. Immunoblotting confirmed the over-expression of *PIK3CA* protein in LAM1:*PIK3CA*^E545^, but not LAM1^EV^ (Fig. 1A). As well, RT-PCR confirmed the expression of the specific PIK3CA^E545K^ mutant variant versus wild type PIK3CA in the altered cells (Additional file 1: Fig. S1). Images of LAM1^EV^ and LAM1:*PIK3CA*^E545K^ cell lines were collected 24 h after plating using brightfield microscopy. Cells demonstrated a less elongated morphology than the LAM1^EV^ cell line, but still similar in overall cell morphology (Fig. 1B). Furthermore, cellular proliferation was significantly increased at both 48 and 72 h for the LAM1:*PIK3CA*^E545K^ cells as compared to control LAM1^EV^ cells using normalized relative fluorescence units (RFU) (*p* < 0.0001) (Fig. 1C).

**Fig. 2:**
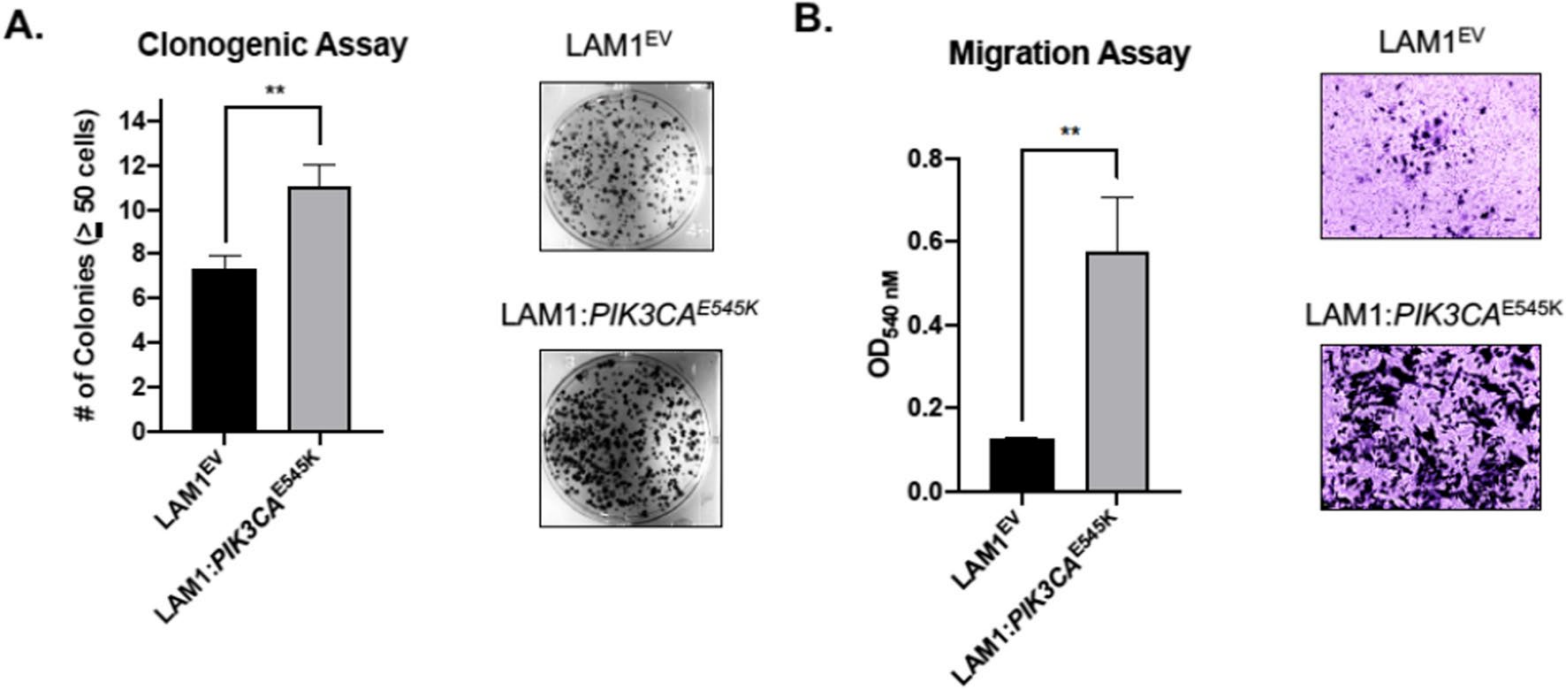
Expression of *PIK3CA*^E545K^ in a *BRAF*-mutant PTC cell line promoted colony formation and cell migration in vitro. **A** Clonogenic assay of LAM1^EV^ and LAM1:*PIK3CA*^E545K^ cells seeded into six-well tissue culture plates at a density of 1,000 cells per well. After 10 days, cells were fixed and stained with 0.5% crystal violet. Brightfield microscopy was used to quantify colony formation, with a colony defined as being ≥ 50 cells. For quantification, three representative fields per well were counted for the number of colonies and this was completed for both the parental and mutant cell lines. **B** Cell migration was quantified using fluorometric analysis. Cells were seeded into the top chamber containing serum-free media at a density of 5 × 10^4^ cells per chamber and each chamber was then placed in a well containing media with serum. Cells which migrated through to serum-containing media after 24 h were quantified using fluorometric detection as described using the standard protocol provided. Fluorescence readings were completed using a microplate reader and representative images were collected using brightfield microscopy

We next measured the impact of expressing an activating *PIK3CA* mutation on clonogenic potential. LAM1:*PIK3CA*^E545K^ demonstrated a significant increase in the number of colonies formed after a two-week period versus the LAM1^EV^ control cells (11.00 ± 1.00 vs. 7.33 ± 0.58; *p* = 0.005 respectively) (Fig. 2A). We also investigated how cell migration was affected by *PIK3CA*^E545K^ using a transwell system and fluorometric analysis and found that cells stably expressing *PIK3CA*^E545K^ demonstrated a significant increase in their capacity to migrate when compared to the control cell line (*p* = 0.004) (Fig. 2B). Together this data suggests that the addition of *PIK3CA*^E545K^ to the LAM1 PTC cell line increased cellular proliferation and promoted colony formation and cell migration, in vitro.

### Expression of *PIK3CA*^E545K^ did not alter cell line sensitivity to etoposide or radiation

**Fig. 3:**
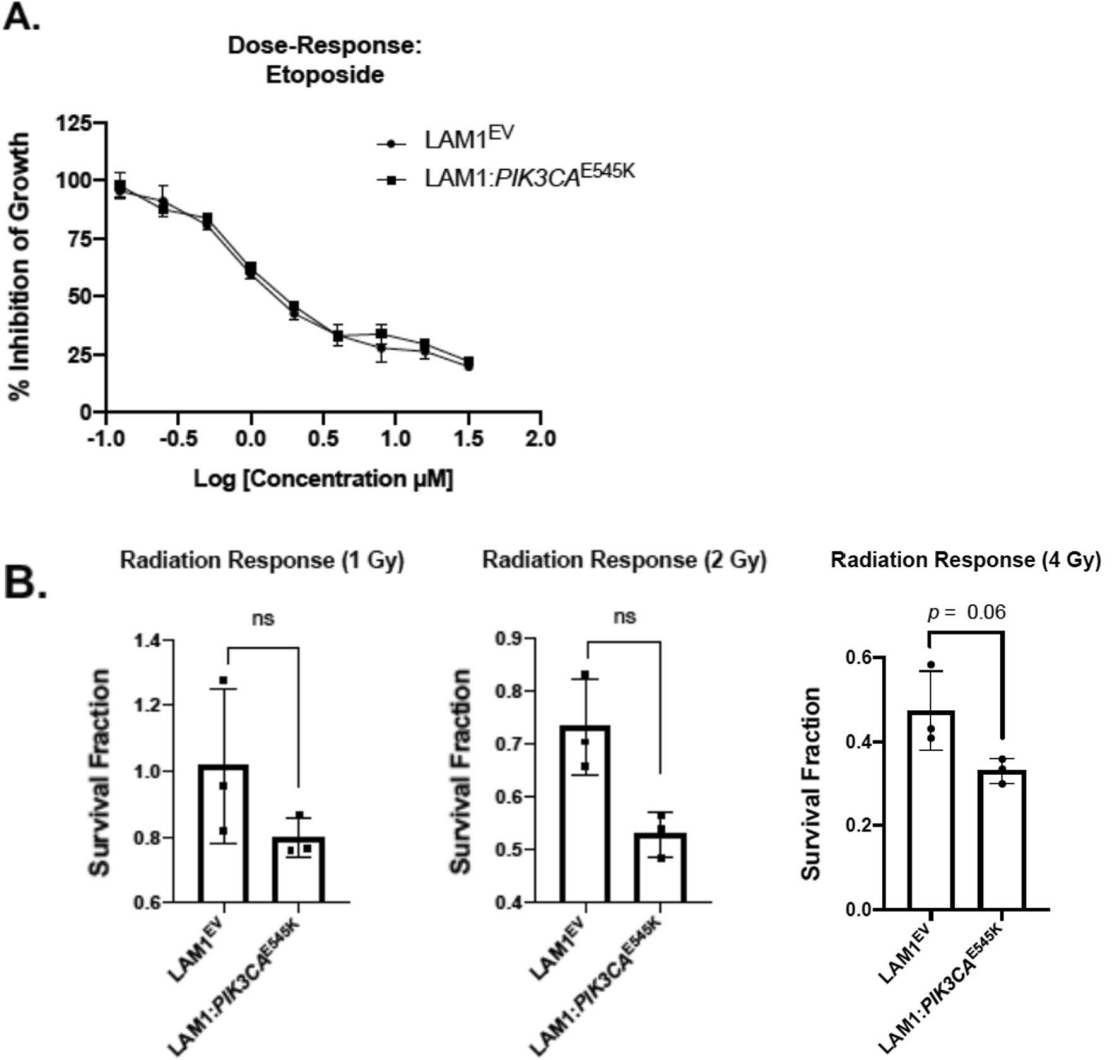
LAM1:*PIK3CA*^E545K^ cells do not exhibit increased resistance to etoposide and radiation in vitro. **A** LAM1^EV^ and mutant cells were seeded into 96-well plates at a density of 2400 cells per well. After 24 h cells were treated with an extended dose range of etoposide concentrations from 0.125 to 32 µM and plate readings were completed following incubation for 72 h. Dose–response curves were generated using Prism 8 GraphPad Software and IC_50_ values were calculated for each cell line. **B** The LAM1^EV^ and LAM1:*PIK3CA*^E545K^ cell lines were seeded at a density of 250 (0 Gy), 500 (1 Gy), 1000 (2 Gy) and 2000 (4 Gy) cells per well, with respective doses of radiation shown in parenthesis applied after 24 h. Cells were incubated for a one-week period with cell media changed every three days. Following this incubation period, cells were fixed and stained with 0.5% crystal violet. Brightfield microscopy was used to visualize colony formation and colonies were quantified, with a colony defined as being ≥ 50 cells. Quantification was completed by calculating the plating efficiency for each control cell line and the respective survival fraction as described by Franken et al*.* [14]

ATC is typically nonresponsive to conventional therapies relative to well-differentiated thyroid cancer subtypes [17, 18]. We therefore chose to investigate potential changes in drug response between LAM1^EV^ and LAM1:*PIK3CA*^E545K^ cells to determine whether the addition of *PIK3CA*^E545K^ to the *BRAF*^V600E^ background resulted in more resistance to clinically used therapy regimes. We first treated cells with etoposide, which is used clinically to treat ATC. We found no significant difference between the responses of the LAM1^EV^ and LAM1:*PIK3CA*^E545K^ cell lines (LAM1^EV^ IC_50_ 0.99 µM vs LAM1:*PIK3CA*^E545K^ IC_50_ 1.02 µM, *p* > 0.05; Fig. 3A). As ATC is radioresistant relative to PTC, we next determined the impact of activated PIK3CA on sensitivity to radiation [4, 17]. We irradiated the cell lines with 1, 2 and 4 Gy. At most radiation doses there was no difference in radiosensitivity between the LAM1^EV^ and LAM1:*PIK3CA*^E545K^ cell lines, except at 2 Gy (*p* = 0.02, Fig. 3B). Collectively, this data suggests that the overall response to conventional therapeutics, which includes treatment with the chemotherapeutic agent etoposide and radiation, did not appear to change dramatically with the introduction of *PIK3CA*^E545K^ in a *BRAF*^V600E^ PTC cell line when tested in vitro.

### Introduction of *PIK3CA*^E545K^ led to loss of well-differentiated thyroid cancer markers in vivo

**Fig. 4:**
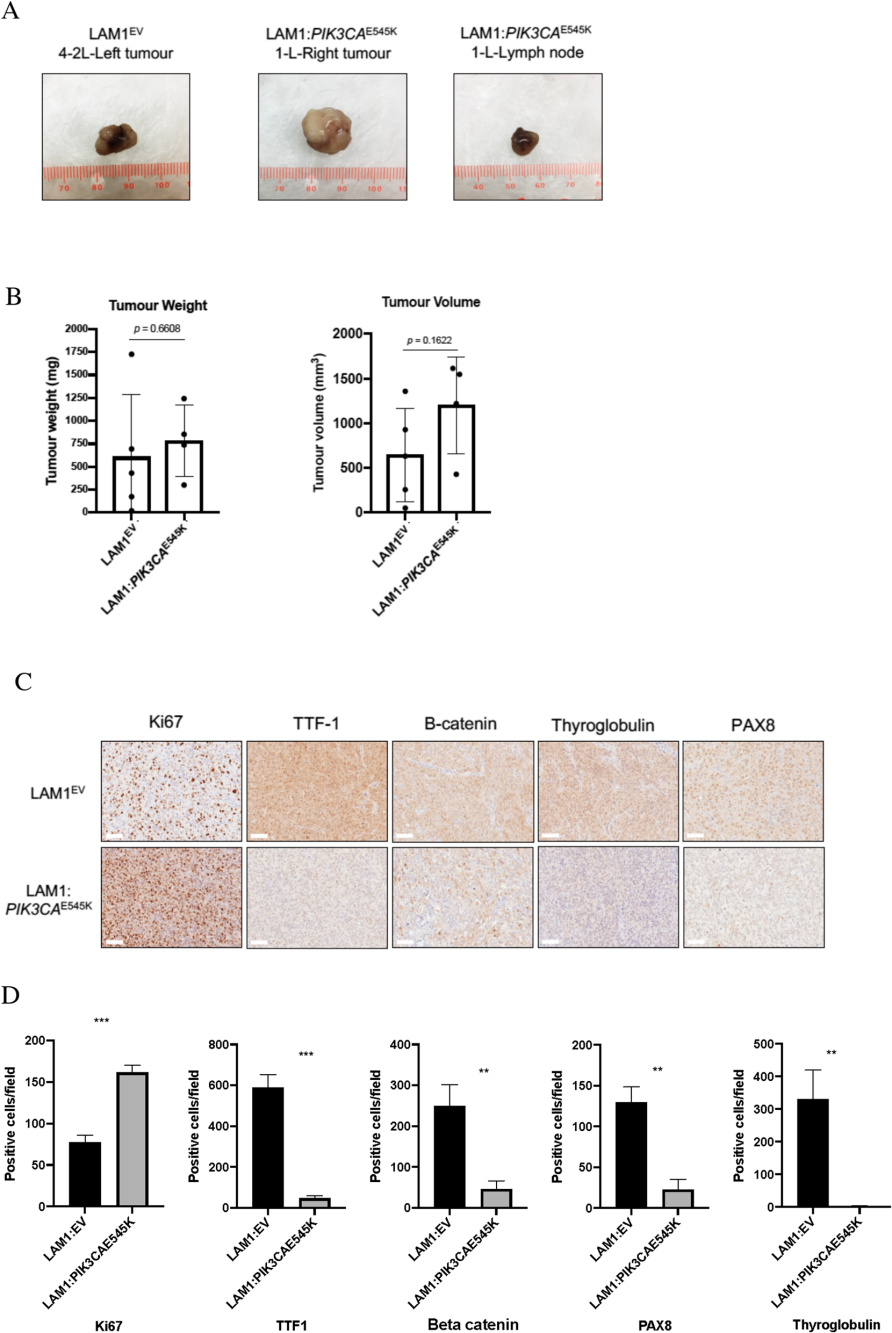
Introduction of *PIK3CA*^E545K^ led to loss of well-differentiated thyroid cancer markers in vivo*.* **A** LAM1^EV^ and LAM1:*PIK3CA*^E545K^ were implanted in the hind flanks of nude mice. Mice were sacrificed at 14 weeks post-injection and tumours were dissected from flanks and stored in formalin for FFPE preparation and IHC. **B** Dissected tumours were weighed (mg) for each specimen and compared between the LAM1^EV^ and LAM1:*PIK3CA*^E545K^ groups. Individual tumour volumes of dissected specimens were calculated using the formula: [length x (width)^2^] × 0.52 and compared between cell line groups. (C,D) Tumours were collected, stained (**C**), and quantified (**D**) to determine relative differences in protein expression of thyroid differentiation markers thyroid transcription factor-1 (TTF-1), PAX8, thyroglobulin and B-catenin between the LAM1^EV^ and LAM1:*PIK3CA*^E545K^ models

Hind flank mouse models were generated using the LAM1^EV^ and LAM1:*PIK3CA*^E545K^ cell lines in order to determine whether there would be any differences in tumour growth, volume and metastatic capacity. In both groups, 2 of 5 mice did not develop detectable tumours over the duration of our 14-week study (Additional file 2: Table S2). Representative images of the dissected tumours from both LAM^EV^ and LAM1:*PIK3CA*^E545K^ groups were generated (Fig. 4A, scale provided in millimetres; mm). The LAM1:*PIK3CA*^E545K^ group, mouse 1-L which had the largest tumour volume, also had an enlarged axillary lymph node located on the upper left side of the mouse (Fig. 4A). This mouse was also found to have the largest tumour volume. Although the average tumour weight and tumour volume were higher in the LAM1:*PIK3CA*^E545K^ versus control xenografts, these differences were not statistically significant (Fig. 4B).

We then utilized IHC to determine whether there were any differences in expression of cellular markers consistent with dedifferentiation of thyroid cells. Ki67 staining was stronger in the LAM1:*PIK3CA*^E545K^ tumours, suggesting increased cellular proliferation in those xenografts (Fig. 4C, D). Well differentiated thyroid cancers almost universally express the markers thyroid transcription factor-1 (TTF-1), PAX8, thyroglobulin and B-catenin; however, loss of expression of these proteins is often characteristic of ATC [17]. While the parental LAM^EV^ cell line expressed all four markers, the edited LAM1:*PIK3CA*^E545K^ cells expressed significantly lower levels of all four markers, suggesting that the tumours expressing activated *PIK3CA* were more poorly differentiated (Fig. 4C, D).

## Discussion

Current evidence suggests that a subset of ATC tumours arise from WDTC through the accumulation of additional mutations [2, 19]. While *BRAF* mutations are common in both WDTC and ATC, *PIK3CA* mutations occur most frequently in ATC and cluster with *BRAF* mutations, suggesting that the simultaneous activation of both pathways represents one route to the genesis of ATC. To test this hypothesis, we stably introduced a *PIK3CA* activating mutation into a *BRAF*-mutant PTC line and assessed the impact on a number of cancer related phenotypes in vitro and in vivo. Activated *PIK3CA* led to an increase in proliferation, clonogenic potential, and migration in vitro, but did not alter sensitivity to etoposide or radiation in vitro. In vivo, cells expressing activated *PIK3CA* did not form tumours that grew faster than the control models; however, they demonstrated loss of markers of well-differentiated thyroid cells, suggesting that the introduction of activated PIK3CA contributed to cellular dedifferentiation. The growth of LAM1 and modified LAM1^EV^ was unexpected as the authors who generated this parental LAM1 cell line reported that cell line xenografts did not engraft or generate tumours in nude mice [11]. STRs confirm that our LAM1 cells are identical to the original parental line and that genetic drift does not explain the difference but may point to inherent differences between mice strain sources or facilities.

Collectively, these findings suggest that activation of *PIK3CA* contributes to at least a portion of the progression of WDTC to ATC. Indeed, *PIK3CA* is just one of several genes found to be more frequently mutated in ATC compared to WDTC. Many other genetic changes are over-represented in undifferentiated thyroid cancer, including mutation of *TP53*, *CDKN2A*, *TERT*, *EIF1AX*; however, many of the variants identified by next generation sequencing studies may be passenger mutations as opposed to cancer drivers [20, 21]. Indeed others have reported that KRAS and PTEN co-mutations are required to drive differentiated thyroid follicular cells into an undifferentiated state [22]. But this is not supported by most deep sequencing studies which clearly show the co-occurrance of BRAF and PIK3CA mutations and suggest that NRAS and PTEN are exclusive (do not occur together) genetic changes at the undifferentiated level [9, 23]. Similar functional validation of these other genes is therefore necessary to determine the context-dependency of these changes. As critical, may be the temporal ordering of the mutations. It may be that de-differentiation is a prerequistite for acquisition of further mutations that can influence proliferation, treatment-resistance or metastases.

To date, ATC has been characterized only by target sequencing or small exome and copy number array studies [9, 10, 23–25]. A larger scale, multi-platform analysis is needed in order to gain a comprehensive understanding of the genetic changes underlying it. Establishing the sequence, copy number, structure/epigenetic and expression landscape of this rare, but lethal disease can further aid in improving our understanding of the progression of WDTC to ATC and can provide the identification of additional variants for validation. Certainly, the rarity of ATC means that curating a large number of high-quality samples is a significant barrier. Large collaborative group efforts are necessary in order to facilitate these important studies.

## Supplementary Information

**Additional file 1: Figure S1**. The expression of activated *PIK3CA* E545K mutant in the stably transfected LAM1 cells was confirmed by RT-PCR. cDNAs prepared from a positive control (PCI6A *PIK3CA* E545K), a specificity control (JHU029 – *PIK3CA* H1047L mutant), and the parental (LAM1) were compared to the LAM1:*PIK3CA* E545K for levels of *PIK3CA* E545K transcripts.

**Additional file 2: Table S1**. Short tandem repeat profiling of LAM1 cell line. The STR profile for both the parental and edited LAM1 cell lines were identical to those reported by Copland et al. [11]. **Table S2**. A hind flank model of disease progression. LAM1EV and LAM1:*PIK3CA*^E545K^ cell lines were used for the generation of cell line xenograft hind flank models of disease (*n* = 5 per group). A total of 1 × 10^6^ cells were injected into the hind flank of each athymic nude mouse. Mice were weighed twice per week and tumour dimensions (length and width) were measured once weekly when tumours were palpable using digital calipers. Tumour weights (mg) were measured and collected after dissection.

## Data Availability

The datasets used and/or analysed during the current study are available from the corresponding author on reasonable request.
