# Supplementary figures and images for: Introduction and expression of PIK3CAE545K in a papillary thyroid cancer BRAFV600E cell line leads to a dedifferentiated aggressive phenotype
Source: J Otolaryngol Head Neck Surg. 2022 Feb 22;51:7. doi: 10.1186/s40463-022-00558-w (PMC8862267; doi:10.1186/s40463-022-00558-w)

## Confirmation of PIK3CA:E545K Expression

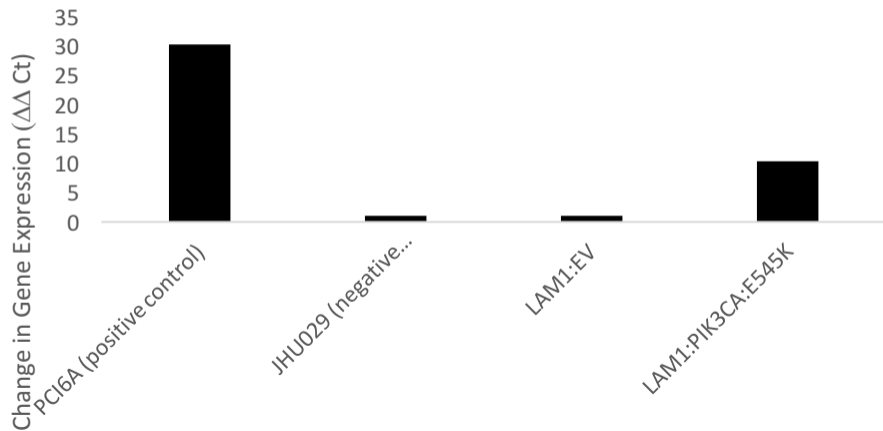

Supplement: Supplementary file 1 — Additional file 1: Figure S1. The expression of activated PIK3CA E545K mutant in the stably transfected LAM1 cells was confirmed by RT-PCR. cDNAs prepared from a positive control (PCI6A PIK3CA E545K), a specificity control (JHU029 – PIK3CA H1047L mutant), and the parental (LAM1) were compared to the LAM1:PIK3CA E545K for levels of PIK3CA E545K transcripts. [file 40463_2022_558_MOESM1_ESM.pdf]
