# Supplementary material for: Introduction and expression of PIK3CAE545K in a papillary thyroid cancer BRAFV600E cell line leads to a dedifferentiated aggressive phenotype
Source: J Otolaryngol Head Neck Surg. 2022 Feb 22;51:7. doi: 10.1186/s40463-022-00558-w (PMC8862267; doi:10.1186/s40463-022-00558-w)
Supplement: Supplementary file 2 — Additional file 2: Table S1. Short tandem repeat profiling of LAM1 cell line. The STR profile for both the parental and edited LAM1 cell lines were identical to those reported by Copland et al. [11]. Table S2. A hind flank model of disease progression. LAM1EV and LAM1:PIK3CAE545K cell lines were used for the generation of cell line xenograft hind flank models of disease (n = 5 per group). A total of 1 × 10^6^ cells were injected into the hind flank of each athymic nude mouse. Mice were weighed twice per week and tumour dimensions (length and width) were measured once weekly when tumours were palpable using digital calipers. Tumour weights (mg) were measured and collected after dissection. [file 40463_2022_558_MOESM2_ESM.docx]

Supplemental Table 1. Short tandem repeat profiling of LAM1 cell line. The STR profile for both the parental and edited LAM1 cell lines were identical to those reported by Marlow et al., 2006.

|  | LAM1 (Copland et al 2006) | | LAM1 | | LAM1:PIK3CA | |
| --- | --- | --- | --- | --- | --- | --- |
| **Marker** | **LAM1** |  | **Allele 1** | **Allele 2** | **Allele 1** | **Allele 2** |
| Amelogenin | X | Y | X | Y | X | Y |
| CSF1PO | 11 | 12 | 11 | 12 | 11 | 12 |
| D13S317 | 10 | 11 | 10 | 11 | 10 | 11 |
| D16S539 | 8 | 12 | 8 | 12 | 8 | 12 |
| D18S51 | 15 | 15 | 15 | 15 | 15 | 15 |
| D19S433 |  |  | 15 | 15 | 15 | 15 |
| D21S11 | ----- |  | 28 | 32.2 | 28 | 32.2 |
| D2S1338 |  |  | 17 | 18 | 17 | 18 |
| D3S1358 | 15 | 17 | 15 | 17 | 15 | 17 |
| D5S818 | 12 | 13 | 12 | 13 | 12 | 13 |
| D7S820 | 10 | 11 | 10 | 11 | 10 | 11 |
| D8S1179 | 12 | 12 | 12 | 12 | 12 | 12 |
| FGA | 19 | 22 | 19 | 22 | 19 | 22 |
| THO1 | 9.3 | 10 | 9.3 | 10 | 9.3 | 10 |
| TPOX | 8 | 8 | 8 | 8 | 8 | 8 |
| vWA | 17 | 19 | 17 | 19 | 17 | 19 |

Supplementary Table 2. A hind flank model of disease progression. LAM1^EV^ and LAM1:*PIK3CA*^E545K^ cell lines were used for the generation of cell line xenograft hind flank models of disease (*n* = 5 per group). A total of 1x10^6^ cells were injected into the hind flank of each athymic nude mouse. Mice were weighed twice per week and tumour dimensions (length and width) were measured once weekly when tumours were palpable using digital calipers. Tumour weights (mg) were measured and collected after dissection.

**
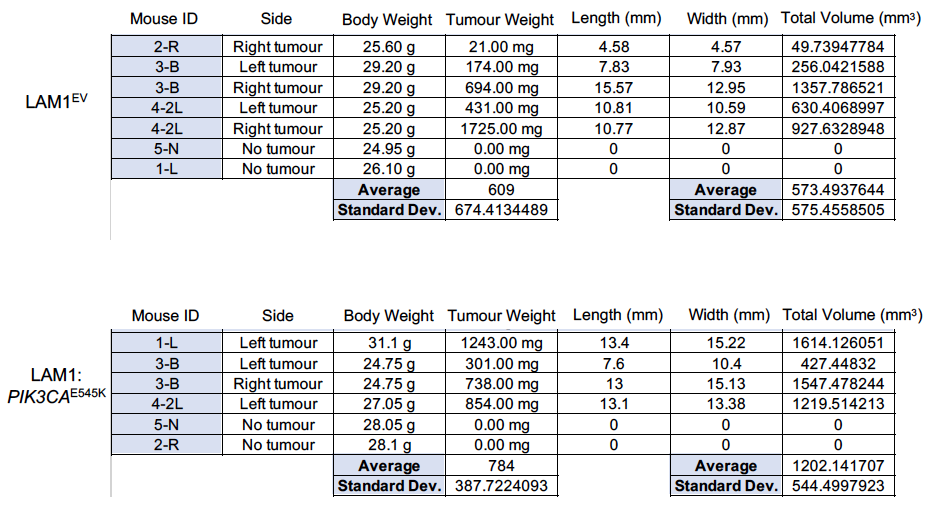
**
